# Male football players have better patient-reported outcomes after primary anterior cruciate ligament reconstruction compared with females

**DOI:** 10.1186/s13102-024-00996-1

**Published:** 2024-09-25

**Authors:** Anne Fältström, Martin Hägglund, Joanna Kvist

**Affiliations:** 1 Region Jönköping County, Rehabilitation Centre, Ryhov County Hospital, 551 85 Jönköping, Sweden; 2 Unit of Physiotherapy, Department of Health, Medicine and Caring Sciences, Linköping University, 581 83 Linköping, Sweden; 3 Sport Without Injury ProgrammE (SWIPE), Department of Health, Medicine and Caring Sciences, Linköping University, 581 83 Linköping, Sweden

**Keywords:** Woman, Sex, Man, Self-reported, Soccer, Return to sport

## Abstract

**Background:**

Sex differences in patient-reported outcomes (PROs) are not well investigated after anterior cruciate ligament (ACL) reconstruction in football players. The aim was to study sex differences in player-related factors, ACL injury characteristics and PROs after primary ACL reconstruction in football players.

**Methods:**

In this cross-sectional cohort study a survey was sent to 390 male and 403 female football players who were injured when playing football and had undergone a primary ACL reconstruction in the previous 1–3 years. Player-related factors, ACL injury characteristics, and PROs covering knee function, satisfaction with activity level and knee function, and readiness to return to sport were compared between male and females. The questionnaires International Knee Documentation Committee Subjective Knee Evaluation Form (IKDC-SKF), Knee injury and Osteoarthritis Outcome Score (KOOS), ACL-Quality of Life (ACL-QoL) and ACL-Return to Sport after Injury (ACL-RSI) were used.

**Results:**

Ninety males (23%) and 283 (70%) females answered the survey, 65 males and 198 females fulfilled the inclusion criteria. Males had returned to football to a higher degree (77% vs 59%, *p* = 0.008) at any time after ACL reconstruction, but at the time of the survey, an equal number of males and females played football (55% vs 47%, *p* = 0.239) and had similar activity level according to the Tegner Activity Score (median, 9; interquartile range [IQR], 7, vs median, 8; IQR, 7; *p* = 0.740). Males were more satisfied with their knee function and activity level and rated higher scores in the IKDC-SKF (mean ± standard deviation, 83 ± 16 vs 76 ± 16, *p* = 0.006), KOOS Sport/Recreation (79 ± 19 vs 72 ± 22, *p* = 0.034), KOOS Quality of Life (73 ± 22 vs 64 ± 20, *p* = 0.008), ACL-QoL (7.6 ± 2 vs 6.8 ± 1.8, *p* = 0.008), and ACL-RSI (6.7 ± 2.1 vs 5.5 ± 2.3, *p* < 0.001) than females (all with small − medium effect sizes).

**Conclusions:**

Male football players reported more favourable results than females in patient-reported knee function, satisfaction with activity level and knee function, knee-related quality of life and psychological readiness to return to sport 1–3 years after ACL reconstruction. The results contribute to a better understanding of the eventual effect of patient sex on outcomes after ACL reconstruction in football players. However, the clinical importance of these differences is unclear.

## Background

The decision to return to football after anterior cruciate ligament (ACL) reconstruction (ACLR) is based on multiple factors and includes both physical status and psychological readiness [1]. Many different factors have a negative impact on return to football, for example, female sex, concomitant cartilage injury, and knee pain [2]. In elite football players, age > 25 years, meniscal surgery at ACLR, and a subsequent surgery after ACLR and before return to football, had negative impacts on the rate of return to football, but no sex differences were observed [3]. Returning to football after ACLR can have both risks and rewards. Female football players who returned to football had higher ratings for the patient-reported outcomes (PROs) knee function, knee-related quality of life and psychological readiness to return to sport [4], but also a doubled risk of sustaining a secondary ACL injury compared with those who did not return [5].

PROs reflect patients’ perspective of their health status and can be used for screening, progress monitoring, and problem identification during rehabilitation and RTS process [6]. Female athletes tend to experience inferior PROs regarding activity and knee-related outcomes after ACLR compared with males [7, 8] and had lower odds of returning to sport (very low-certainty evidence) within the first 5 years after ACLR [8]. Female football players with ACLR experienced a higher incidence of secondary ACL injury compared with males (27% vs 10%), but sex differences in PROs are not well investigated after ACLR in football players [9]. Thus, the present study aimed to investigate potential sex differences in player-related factors, ACL injury characteristics, and PROs covering knee function, satisfaction with knee function and activity level, and readiness to return to sport in male and female football players 1–3 years after unilateral primary ACLR.

## Methods

### Participants

Participants for this cross-sectional study were identified through the Swedish National Knee Ligament Register (SNKLR) [10] in three regional football districts geographically located near Linköping University in Sweden and for the females also via advertisements on the websites of the same districts. The inclusion procedure for female football players has been described in detail previously for the purpose of evaluating differences between females who had returned or had not returned to football [4] and regarding risk factors for sustaining a secondary knee injury [5, 11]. The inclusion criteria for this analysis were age 16–25 years, active football player at the time of the primary ACL injury, and having undergone a primary ACLR in the previous 1–3 years. Exclusion criteria were an associated posterior cruciate ligament injury and/or surgically treated injuries to the medial or lateral collateral ligament, re-rupture of the ACL graft, a contralateral ACL injury, or no response to any of the PROs. Information about the ACLR procedure and any concomitant surgically treated meniscus or cartilage injuries at ACLR was extracted from the SNKLR. Data were collected in the football pre-season period (January–April) in 2013, 2014 and 2015 for the females and 2018 for the males. The players were contacted via mail with information about the study and login details for a web-based questionnaire. Non-responders were sent up to 3 reminders. Patient-reported data were obtained through a battery of questionnaires that took approximately 20 min to complete [4]. The study adhered to the Declaration of Helsinki, all participants were given written information about the study, signed a written informed consent form before inclusion, and the study was approved by the Swedish Ethical Review Authority (Dnr 2012/24–31, 2013/75–32, 2017/450–32) and by the SNKLR board.

### Data collection

#### Player-related factors

Demographic data (age, height, weight, family history of ACL injury, smoking) and football-related factors included playing position, preferred kicking leg, and level of play divided into elite (2 top divisions in Sweden), sub-elite, and recreational level (2 lowest divisions and youth play) [4]. Participants were asked to rank the reasons for playing football before ACLR by importance with the fixed response options “to win, practice/prepare for competition, have fun, help the team, health reasons, satisfy other or other reasons (please specify) [4, 12] and risk behaviour during football before ACLR with responses on a 4-point scale scored from “avoid risks at any price” to “often take deliberate risks” [4, 12, 13]. The players stated if they had returned to football after ACLR, if they still played at the time of follow-up and eventual reasons for not playing with the fixed response options “poor knee function, do not trust the knee, fear of new injury, lack of time because of family/work/studies, not fun, change in team or coach, other reasons (please specify) [4, 5, 14]. Participants reported their current activity and participation frequency (times/week) and the first author graded the activity level according to the Tegner Activity Score [15, 16].

#### ACL injury characteristics

ACL injury-related information included injury mechanism (contact or non-contact), time between injury and ACLR, duration of supervised rehabilitation before and after the ACLR, and appraisal of the importance of the physiotherapist contact for their knee function rated on a 5-point scale from “necessary for my current knee function” to “not necessary at all” [4].

#### Patient-reported outcomes

Satisfaction with their current activity level was rated on a scale ranging from 1 (not satisfied at all) to 10 (very satisfied) [14, 16]. Satisfaction with knee function was measured by responses to the question, “If you had to live with your current knee function for the rest of your life, would you feel…”; the responses (delighted, pleased, mostly satisfied, mixed, mostly dissatisfied, unhappy and terrible) were graded on a 7-point scale [16–18]. The following standardized questionnaires were used to evaluate knee function, knee-related quality of life, and readiness to return to sport: International Knee Documentation Committee Subjective Knee Evaluation Form (IKDC-SKF) [19–21], Knee injury and Osteoarthritis Outcome Score (KOOS) subscales Symptoms, Pain, Sport/Recreation and Quality of Life, all ranging from 0 (worse) to 100 (best) [22], ACL-Quality of Life (ACL-QoL) [23, 24], and ACL-Return to Sport after Injury (ACL-RSI) [13, 25], both ranging from 1 (worse) to 10 (best). These instruments evaluated for patients with ACLR have an acceptable internal consistency and test–retest reliability (Cronbach's α > 0.70), and demonstrate evidence of sufficient divergent construct validity [13, 23, 24, 26–28]. Scores for patient-acceptable symptom state 1 to 5 years after ACLR were 75.9 points for IKDC-SKF, 75.0 points for KOOS Sport/Recreation and 62.5 points for KOOS Quality of Life [29].

### Statistical analysis

All statistical analyses were performed using IBM SPSS Statistics for Windows (version 27.0; IBM Corp; Armonk, NY). Means ± standard deviation (SD) or median and interquartile range (IQR) were calculated for descriptive statistics depending on the data level and normality. Numbers and percentages of players reaching a patient-acceptable symptom state in IKDC-SKF, KOOS Sport/Recreation and KOOS Quality of Life were calculated. Between-group comparisons were made between males and females, and for dropout analysis between responders and non-responders separately for males and females, using Student’s* t* test (ratio data with normal distributions), Mann–Whitney *U* test (ordinal data or non-normal distributions), chi-square test and Fisher’s exact test (nominal data) as appropriate. Effect sizes with Cohen’s *d* (limits: 0.2, small effect; 0.5, medium effect; 0.8, large effect) were calculated. Cohen’s *d* values were transformed from *η*^2^ when using the Mann–Whitney *U* test. The level of significance was set at *p* < 0.05.

## Results

**Fig. 1:**
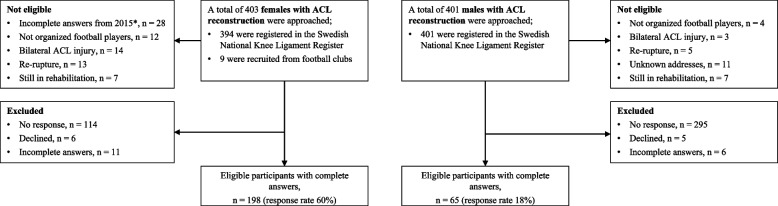
Participant flow diagram. *In the main study (a prospective cohort study), the aim was to find active football players for the ongoing prospective study about risk factors and therefore 28 players (in 2015) who had quit football did not answer the entire survey

Two hundred and sixty-three participants were included in the analyses (65 males and 198 females) (Fig. 1). The mean follow-up after ACLR was 2.2 ± 0.6 years for males and 1.8 ± 0.6 years for females (*p* < 0.001) (Table 1).

**Table 1:** Player-related factors in male and female football players after primary ACL reconstruction

	Males (*n* = 65)	Females (*n* = 198)	*p* value
Age at survey (years), mean (SD)	21.1 (2.7)	20.4 (2.7)	0.077
Age at ACLR (years), mean (SD)	19.0 (2.5)	18.6 (2.6)	0.306
Time from ACLR to follow-up (years), mean (SD)	2.2 (0.6)	1.8 (0.6)	** < 0.001**
1–2 years, *n* (%)	28 (43)	116 (59)	**0.029**
> 2 years, *n* (%)	37 (57)	82 (41)	
Height (cm), mean (SD)	181.1 (6.1)	167.4 (5.7)	** < 0.001**
Weight (kg), mean (SD)	76.5 (9.3)	62.9 (7.9)	** < 0.001**
Body mass index (kg/m^2^), mean (SD)	23.3 (2.3)	22.5 (2.5)	**0.019**
Immediate family with ACL injury, *n* (%)	14 (22)	69 (35)	**0.045**
Occupation, *n* (%)			
Worker	30 (46)	69 (35)	0.103
Student	35 (54)	129 (65)	
Smoker, *n* (%)			
No	64 (97)	189 (96)	0.271
Yes	1 (2)	9 (5)	
Level of play before ACL injury, *n* (%)			
Elite	4 (6)	10 (5)^a^	0.923
Sub-elite	50 (77)	149 (76)	
Recreational	11 (17)	36 (19)	
Playing position before ACL injury, *n* (%)			
Goalkeeper	5 (8)	9 (5)^a^	0.374
Defender	29 (45)	72 (37)	
Midfield	19 (29)	78 (40)	
Forward	12 (19)	36 (19)	
Preferred kicking leg, *n* (%)			
Right or both	58 (89)	180 (92)^a^	0.440
Left	7 (11)	15 (8)	
Most important reason for playing football before the ACL injury (retrospective assessment), *n* (%)			** < 0.001**
Have fun	23 (35)	117 (59)	
To win	21 (32)	26 (13)	
Practice/prepare for competition	11 (17)	38 (19)	
Health reasons	4 (6)	6 (3)	
Other reasons (e.g. help the team, passion, lifestyle)	6 (9)	11 (6)	
Risk behaviour before ACL injury (retrospective assessment), *n* (%)			
1 Avoided risks at any price	2 (3)	1 (1)^b^	0.246
2 Tried to avoid risks most of the time	20 (31)	52 (26)	
3 Sometimes took deliberate risks	31 (48)	94 (48)	
4 Often took deliberate risks	12 (18)	50 (25)	
Returned to football after ACL reconstruction, *n* (%)	50 (77%)	116 (59%)	**0.008**
Still playing football, *n* (%)	36 (55%)	93 (47%)	0.239
Reasons for not playing football, *n* (%)			**0.015**
Poor knee function,	4 (14)	20 (19)	
Do not trust the knee	4 (14)	20 (19)	
Fear of new injury	2 (7)	24 (23)	
Lack of time	7 (24)	8 (8)	
Not fun	3 (10)	5 (5)	
Change in team or coach	2 (7)	18 (17)	
Other reasons (e.g. performed other sports, moved, other priorities)	7 (24)	10 (10)	
Current activity level, Tegner Activity Score (0–10), median (IQR)	9 (7)	8 (7)	0.740

*Abbreviations* *ACL* Anterior cruciate ligament, *SD* Standard deviation, *IQR* Interquartile range

*p* values in bold type are significant

^a^3 missing values

^b^1 missing value

Data for the non-responders for males and females (time between injury and ACLR, time from ACLR to follow-up, graft, presence of concomitant injuries at ACLR) showed a difference only for age at ACLR for males; the non-responders were slightly older at ACLR than responders 19.9 ± 2.6 vs 19.1 ± 2.5, *p* = 0.015.

### Player-related factors

Males had returned to football to a higher degree than females at any time after ACLR (77% vs 59%), but an equal number of males and females played football at the time of the survey (55% vs 47%). Males stated that the most important reason for playing football before ACL injury was to “have fun” (35%) and to “win” (32%) compared with females (59% and 13%, respectively). Males quit football due to poor knee function, not trusting the knee, and fear of new injury less often than females (35% vs 61%) (Table 1).

### ACL injury characteristics

Males had more lateral meniscus injuries (32% vs 19%, *p* = 0.028) and cartilage injuries (5% vs 1%, *p* = 0.048) at primary ACLR that required surgery (resection, suture, microfracture) compared with females. No other ACL injury-related factors differed significantly between the groups (Table 2).

**Table 2:** ACL injury-related factors in male and female football players after ACLR

ACL injury-related factors	Males (*n* = 65)	Females (*n* = 198)	*p* value
**Injury and surgical factors**			
Age at injury (years), mean (SD)	18.3 (2.4)	18.0 (2.7)^a^	0.339
Injury mechanism, *n* (%)			
Contact	22 (58)^b^	79 (42)^c^	0.068
No contact	16 (42)	110 (58)	
Time between injury and ACLR (days), median (IQR)	183 (159)^a^	165 (152)	0.125
0–90 days, *n* (%)	8 (13)	48 (24)	0.137
91–365 days, *n* (%)	45 (70)	121 (61)	
> 365 days, *n* (%)	11 (17)	29 (15)	
Graft, all autografts, *n* (%)			
Hamstrings (semitendinosus and semitendinosus-gracilis)	62 (95)	190 (96)	0.591
Patellar tendon	1 (2)	5 (3)	
Quadriceps	2 (3)	2 (1)	
Other	0 ()	1 (1)	
ACLR knee, *n* (%)			
Right	36 (55)	100 (51)	0.495
Left	29 (45)	98 (49)	
Presence of concomitant injuries at ACLR, *n* (%)			
Meniscus injury (medial)	13 (20)	35 (18)	0.674
Meniscus injury (lateral)	21 (32)	38 (19)	**0.028**
Articular cartilage injury	3 (5)	1 (1)	**0.048**
**Rehabilitation factors**			
Physiotherapist contact before ACLR, *n* (%)			
Yes	48 (74)	167 (84)	0.057
< 3 months	19 (40)	41 (25)	0.082
3–6 months	18 (38)	57 (34)	
6–9 months	5 (10)	26 (16)	
> 9 months	6 (12)	43 (26)	
Physiotherapist contact after ACLR, *n* (%)			
Yes	63 (97)	194 (98)	0.621
< 3 months	2 (3)	4 (2)	0.687
3–6 months	16 (25)	43 (22)	
6–9 months	22 (35)	60 (31)	
> 9 months	24 (37)	87 (45)	
Appraisal of the physiotherapist contact, *n* (%)			
Necessary for the current knee function	51 (78)	153 (77)	0.843
Necessary to some extent	11 (17)	33 (17)	
Neutral	2 (3)	7 (3)	
Not very necessary	1 (1)	2 (1)	
Not necessary at all	2 (2)	1 (0)	
No physiotherapist contact	0 (0)	2 (1)	

*Abbreviations ACL* Anterior cruciate ligament, *ACLR* Anterior cruciate ligament reconstruction, *IQR* Interquartile range, *SD* Standard deviation

*p* values in bold type are significant

^a^1 missing value

^b^27 missing values

^c^9 missing values

### Patient-reported outcomes

Males were more satisfied with their knee function and current activity level and rated higher scores in IKDC-SKF, KOOS subscales Sport/Recreation and Quality of Life, ACL-QoL, and ACL-RSI compared with females (Table 3).

**Table 3:** Patient-reported outcomes (knee function, satisfaction with knee function and activity level, and readiness to return to sport) in male and female football players after ACL reconstruction

	Males (*n* = 65)	Females (*n* = 198)	Mean difference (95% CI)	*p* value	Cohen’s *d*
Satisfied with current activity level (1–10), median (IQR)	8 (3)	7 (4)		**0.019**	0.31
Satisfaction with knee function (1–7), median (IQR)	2 (1.5)	3 (2)^a^		**0.010**	0.31
Delighted (1), *n* (%)	15 (23)	27 (14)			
Pleased (2), *n* (%)	22 (34)	55 (28)			
Mostly satisfied (3), *n* (%)	12 (19)	39 (20)			
Mixed feelings (4), *n* (%)	11 (17)	46 (23)			
Mostly dissatisfied (5), *n* (%)	2 (3)	10 (5)			
Unhappy (6), *n* (%)	2 (3)	11 (6)			
Terrible (7), *n* (%)	1 (2)	10 (5)			
IKDC-SKF (0–100), mean (SD)	82.8 (16.1)	76.4 (16.0)^c^	6.3 (1.8 − 10.8)	**0.006**	0.39
IKDC-SKF, patient-acceptable symptom state ≥ 75.9, *n* (%)	51 (79)	109 (55)		** < 0.001**	
KOOS (0–100), mean (SD)					
Symptoms	78.7 (15.3)^b^	78.1 (16.3)	0.6 (− 4.3 to 5.5)	0.806	0.04
Pain	87.7 (14.6)	86.2 (13.0)	1.5 (− 2.5 to 5.6)	0.458	0.11
Sport/Recreation	78.8 (18.9)	71.6 (22.1)	7.1 (0.5 − 13.7)	**0.034**	0.33
Sport/Recreation, patient-acceptable symptom state ≥ 75.0, *n* (%)	36 (69)	106 (55)		0.064	
Quality of life	73.1 (22.1)	64.5 (20.1)	8.6 (2.3 − 14.9)	**0.008**	0.42
Quality of life, patient-acceptable symptom state ≥ 62.5, *n* (%)	40 (77)	124 (64)		0.077	
ACL-QoL (1–10), mean (SD)	7.6 (2.0)	6.8 (1.8)		**0.008**	0.41
ACL-RSI (1–10), mean (SD)	6.7 (2.1)^d^	5.5 (2.3)		** < 0.001**	0.53

*Abbreviations ACL* Anterior cruciate ligament, *ACL-QoL* Anterior Cruciate Ligament-Quality of Life, *ACL-RSI* Anterior Cruciate Ligament-Return to Sport after Injury scale, *CI* Confidence interval, *IQR* Interquartile range, *IKDC-SKF* International Knee Documentation Committee Subjective Knee Evaluation Form, *KOOS* Knee injury and Osteoarthritis Outcome Score, *SD* Standard deviation

Cohen’s *d* values were transformed from *η*^2^ with effect size limits: 0.2, small effect; 0.5, medium effect; 0.8, large effect. *p* values in bold type are significant

^a^3 missing values

^b^12 missing values

^c^4 missing values

^d^2 missing values

## Discussion

The most important findings of the present study were that males were generally more satisfied with their activity level and knee function than females. Males had returned to football to a higher degree, but at the time of the survey, an equal number of males and females still played football and had the same activity level.

Males were more satisfied with their activity level and knee function, rated higher scores for knee function and knee-related quality of life (IKDC-SKF, KOOS subscales Sport/Recreational and Quality of Life, and ACL-QoL) and psychological readiness to return to sport (ACL-RSI). These sex differences are in line with previously reported results in general populations who underwent ACLR. In a systematic review and meta-analysis on sex-specific outcomes after ACLR, males had higher postoperative scores for IKDC-SKF compared with females, but the mean difference was only 3 points [7]. Another systematic review reported that male athletes tend to experience superior PROs regarding knee-related outcomes such as IKDC-SKF (2 points), KOOS subscales Sport/Recreation (10 points), and KOOS Quality of Life (2‒5 points) within the first 5 years after ACLR compared with females [8]. However, the clinical relevance of these reported differences in PROs may be questioned [30]. In our cohorts, more males than females reached previously published patient-acceptable symptom states [29] for IKDC-SKF (79% vs 55%), but there were no differences for KOOS Sport/Recreation and Quality of Life. Thus, the clinical relevance of the differences detected in the PROs is unclear.

Important ACL injury characteristics that could influence knee function, quality of life and satisfaction with knee function and activity level, such as time between injury and ACLR, graft type, age at injury, age at ACLR, contact or non-contact injury mechanism, and contact with physiotherapist did not differ between males and females. Males, compared with females, had more lateral meniscus injuries (31% vs 19%) and cartilage injuries (5% vs 1%) at primary ACLR that required surgery. Concomitant meniscus and cartilage injuries are prognostic factors for worse long-term PROs after ACLR [31]. However, the males in our study had favourable PROs compared with females even though they had more lateral meniscus and cartilage injuries. Otherwise, the reasons for the favourable results for male football players are unclear. Generally, males tend to report better scores for overall quality of life, physical and psychological symptoms, and emotional function compared with females [32]. A possible explanation for the favourable results for the male football players is potential sex differences in socioenvironmental factors. Gender, age, and level of sports participation can influence factors such as independence, recovery expectations, social support, engagement in care, environmental influences, and sport culture factors, and influence the recovery following ACLR [33]. Further studies are needed to evaluate sex differences in outcomes after ACLR in football players to broaden the knowledge in this area and to be able to give all patients better advice on expected results.

Males and females played football to a similar degree (55% and 47%, respectively) at the time of the survey, but more males reported that they had returned to football after the ACLR (77% vs 59%). However, the mean follow-up after ACLR differed slightly (2.2 ± 0.6 years for males and 1.8 ± 0.6 years for females), which partly can explain why more males had returned to football. Mean time to return to football has been reported to be 9 months after ACLR [34]. So, it is likely that two in every five male players only played for one season after ACLR and quit playing again shortly after their return to football. Previous reports suggest that males generally return to football to a higher degree (60% − 76%) than females (46% − 67%) [2, 35]. Return to football is mostly reported using a yes/no question and sometimes also the level of football play after ACLR, but rarely with information about how long the career lasts [2, 35]. Therefore, such reports could be misleading in that career longevity may also be a factor in a successful return to sport. In our study, males and females had similar high activity levels according to Tegner Activity Score (median, 9 vs 8). This is in contrast to previous findings where females exhibited inferior Tegner Activity Scores at most time points after ACLR than males [8]. Previously, athletes and football players who did not return to sport after ACLR reported lower scores in PROs [4, 9, 36]. Our players had similar activity level suggesting that the sex differences found in PROs were not contingent on whether they had returned to football or not.

Males more often stated “to win” compared to females as an important reason for playing football before the ACL injury. Sport performance, fun, and well-being as motives for participation in sport predicted poor KOOS scores, especially for the subscale Sport/Recreation 2 years after ACL injury even after adjusting for sex and age, for example [37]. Thus, the reasons for playing football may be an important factor to consider in relation to PROs after ACLR.

### Strengths and limitations

One strength of the present study is the homogeneity of the study sample regarding factors that could have an impact on PROs. We excluded players who reported new ACL injuries because it is well known that additional ACL injuries lower the activity level and PROs [38]. Our study has some limitations. First, we expected to have a similar response rate among the male football players as for the females and a post-hoc analysis could have been done. However, previously performed sample size calculations with patients with ACLR indicated that 42 and 56 participants in each group were needed, based on clinically relevant difference of 11.5 in IKDC-SKF [39] and 8 points in KOOS subscale quality of life [40], with a power of 80% and a confidence interval of 95%. Thus, the most important limitation in our view is the risk of selection bias due to the low response rate, especially for the males, and this could impact the generalizability of our results. A previous study showed lower response rates for male compared with female football players [2]. We did a response analysis and the non-responders (males) only differed in age at ACLR (less than 1 year older). We had an acceptable response rate for the females (60%). We wanted to make the data collection as similar as possible for the males, but there were some minor modifications. We advertised only for the females to eventually recruit players not registered in the SNKLR. However, this only resulted in 9 extra female players and would probably not affect the results. There was a slight difference in mean follow-up time that could have affected the results regarding return to football after ACLR; males had a few months more after ACLR before answering the survey compared with females. The data for the males were collected 3‒5 years after the females, because our initial design was to evaluate female football players who had returned or had not returned to football [4]. However, the surgical techniques [10], rehabilitation after ACLR, and football play (intensity) did not change significantly during these few years. Another limitation is that the questions about reasons for playing or not playing football are not validated. However, participants had the opportunity to provide reasons through free text answers.

## Conclusions

We observed sex differences in patient-reported knee function, satisfaction with activity level and knee function, knee-related quality of life and psychological readiness to return to sport 1–3 years after ACLR. Male football players reported more favourable results than females. These results contribute to a better understanding of the eventual effect of patient sex on outcomes after ACL reconstruction in football players. However, the clinical importance of these differences is unclear.

## Data Availability

De-identified data are available from the first author (AF) upon reasonable request.

## Abbreviations

ACL: Anterior Cruciate Ligament
ACLR: Anterior Cruciate Ligament Reconstruction
ACL-RSI: Anterior Cruciate Ligament Return to Sport after Injury
ACL-QoL: Anterior Cruciate Ligament Quality of Life
CI: Confidence Interval
IKDC-SKF: International Knee Documentation Committee Subjective Knee Evaluation Form
KOOS: Knee injury and Osteoarthritis Outcome Score
SPSS: Statistical Package for Social Sciences
